# Paracoccidioidomycosis and pregnancy: A 40-year single-center cohort study in the endemic area of Rio de Janeiro, Brazil

**DOI:** 10.1371/journal.pntd.0011645

**Published:** 2023-09-14

**Authors:** Lorena Macedo Pestana Benko, Mariana Evangelista de Souza Vieira da Silva, Eduardo Mastrangelo Marinho Falcão, Dayvison Francis Saraiva Freitas, Guilherme Amaral Calvet, Marcos de Abreu Almeida, Rodrigo Almeida-Paes, Rosely Maria Zancopé-Oliveira, Antonio Carlos Francesconi do Valle, Priscila Marques de Macedo

**Affiliations:** 1 Laboratory of Clinical Research on Infectious Dermatology, Evandro Chagas National Institute of Infectious Diseases, Fiocruz, Rio de Janeiro, Brazil; 2 Acute Febrile Illnesses Laboratory, Evandro Chagas National Institute of Infectious Diseases, Fiocruz, Rio de Janeiro, Brazil; 3 Mycology Laboratory, Evandro Chagas National Institute of Infectious Diseases, Fiocruz, Rio de Janeiro, Brazil; Albert Einstein College of Medicine, UNITED STATES

## Abstract

The occurrence of acute paracoccidioidomycosis (PCM) in urban areas of the Rio de Janeiro state, Brazil, has emerged in recent years. Therefore, young populations, including pregnant women, are at a higher risk of infection. Furthermore, young women undergoing itraconazole treatment for PCM have increased chances to get pregnant because this medication may reduce the effectiveness of contraceptives. Acute PCM is invasive, reaching abdominal organs, posing a maternal-fetal risk. PCM treatment in pregnant women is also challenging due to the teratogenicity associated with the currently available oral drugs. There are scarce studies on PCM and pregnancy, mainly consisting of case reports and experimental murine models that highlight the severity of this association. We conducted a database research at a PCM reference center in Rio de Janeiro state from 1980 to 2020. We included patients diagnosed with PCM who were pregnant shortly before, at admission, or at any moment of their PCM follow-up care. Data related to pregnancy, childbirth, and the newborn were obtained from the Brazilian official public databases. We also reviewed the epidemiological and clinical features of these patients. During the study period, we identified 18 pregnant patients, with a median age of 26 years (range: 16–38). Among these cases, six (33.3%) were detected in the last 5 years, and 14 (77.8%) presented acute PCM, supporting the recent shift in the epidemiological profile towards acute PCM. Most pregnancies occurred during PCM treatment (n = 11, 61.1%), which led to challenges in the therapeutic management. Maternal-fetal complications occurred in some of these cases, including vaginal bleeding (n = 1), preeclampsia (n = 1), prematurity (n = 2), low birth weight (n = 4), and fetal deaths (n = 2). PCM during pregnancy presents a significant public health concern in the context of the emergence of acute PCM in urban areas.

## Author summary

Paracoccidioidomycosis (PCM) is a fungal infection that is common in Brazil and other parts of Latin America. There are two clinical forms: the chronic, which affects rural workers and the acute, which is usually more severe and tends to affect young people. The Rio de Janeiro state, Brazil, is endemic for PCM, and juvenile cases have increased in the recent years. This is concerning for pregnant women since they could also be affected. In addition, young women could get pregnant during the PCM treatment because itraconazole, the main medication to treat PCM, may reduce the effectiveness of contraceptives. This could represent a risk to both mother and fetus because acute PCM is more invasive, reaching abdominal organs. PCM treatment in pregnant women is a challenge because the available oral drugs to treat this mycosis can be harmful to the fetus. There is little information about this important issue in the literature. Therefore, we investigated cases of PCM in pregnancy performing a search in the databases of a PCM specialized center in Rio de Janeiro state. We found 18 pregnant patients in the period of 1980–2020, most of them presenting acute PCM (n = 14, 77.8%) with a moderate to severe clinical profile. Most pregnancies occurred during PCM treatment (n = 11, 61.1%), which created difficulties in the management of both conditions. Some of these cases experienced complications, including prematurity, low birth weight, and two fetal deaths. PCM in pregnancy is an important public health issue and the authors hope to contribute with the knowledge of this subject, to improve care for this vulnerable population.

## Introduction

Paracoccidioidomycosis (PCM) is the main endemic systemic mycosis in Latin America, with Brazil being the most affected country. PCM is caused by pathogenic fungi of the *Paracoccidioides* genus, which are present in soil with high moisture levels in endemic regions. The infection occurs through inhalation of conidia and other infective fungal propagules, usually after engaging in activities involving soil manipulation [1].

PCM manifests in two main clinical forms: chronic (the classic adult type) and acute (the juvenile type). The former comprises approximately 90% of cases and typically affects middle-aged men, mainly rural workers. After a long latency period, the lungs and the upper aerodigestive mucosa are the most affected organs. On the other hand, acute PCM accounts for approximately 10% of cases, and affects the phagocytic mononuclear organs of children and young adults of both sexes. These individuals are genetically susceptible to *Paracoccidioides* spp., usually resulting in a more invasive, disseminated, and severe clinical presentation of this fungal disease [1, 2].

In recent decades, there have been notable shifts in the PCM epidemiology due to human activities that disturb the environment. Rampant deforestation for pastureland and soybean agriculture contributes to climate and precipitation changes, as well as alterations in the rural labor practices, inducing important migratory flows across the country [3]. Additionally, large-scale constructions have been associated with the emergence of acute PCM cases in non-traditional areas associated with this neglected mycosis [3–5].

The Rio de Janeiro state, Brazil is considered an endemic PCM region and has gained increasing epidemiological importance in recent years due to anthropogenic actions that have led to the emergence of acute PCM in urban areas of this state [6,7]. Therefore, young vulnerable populations, including pregnant women, are at a higher risk of infection. Moreover, young women undergoing the long-term treatment for PCM with itraconazole have an increased likelihood of getting pregnant since this drug may reduce the effectiveness of contraceptive drugs [8]. This is a worrisome and relevant problem since acute PCM is more invasive, and can affect lymph abdominal organs, which may represent risks to both mother and fetus.

PCM can occur during pregnancy either as an initial manifestation of the mycosis or as a reactivation of a latent fungal infection. This may be due to the reduced immune response that takes place during gestation, which also predisposes to other systemic mycoses [2]. There is a lack of comprehensive research on PCM and pregnancy in the literature. Most studies consist of case reports that highlight the severity of this association [9–14]. Among these studies, four reported cases of acute PCM, with one case associated with AIDS, presenting ocular and neurological involvement [10]. Preterm birth occurred in three cases [9,10,12] and in one of these cases, the mother, who had no comorbidities reported, died twenty days after delivery [12]. Placental involvement was observed in one case by histopathology study, without complications for the newborn [9].

Difficulties in the diagnosis and treatment of PCM were frequent in the cases above mentioned. Indeed, the treatment of PCM during pregnancy is challenging due to the teratogenicity of many drugs available to treat this fungal disease. Although itraconazole and sulfamethoxazole/trimethoprim (both category C) [15] are the main oral drugs recommended to treat PCM, they should be avoided during pregnancy due to the possibility of teratogenic effects [2]. However, a systematic review and meta-analysis previously reported the safety of cotrimoxazole during pregnancy [16]. In addition, although classified as category C, sulfadiazine is an alternative option according to the Brazilian guidelines for the clinical management of PCM, but must be discontinued 15 days prior to the expected delivery date to avoid kernicterus [2]. Amphotericin B (category B), usually recommended to treat severe cases of PCM, can be used at any moment of the pregnancy [2,17]. After delivery, patients should receive antifungal treatment, preferably with sulfamethoxazole/trimethoprim, which can be used during the breastfeeding [2]. Subsequently, the usual drugs are prescribed until the patients meet the cure criteria [2,17].

Experimental studies conducted on pregnant murine models infected with *Paracoccidioides* spp. revealed an increased occurrence of abortions, newborns with low birth weight and small for gestational age [18]. However, these studies did not provide evidence of placental transmission of PCM to the fetus. Furthermore, pregnant animals exhibited a more severe fungal disease compared to non-pregnant animals [18,19].

Given the importance and limited availability of information on this subject in the current literature, the authors conducted a study to evaluate the PCM occurrence in pregnant women at a reference center for infectious diseases in Rio de Janeiro state, Brazil, aiming to contribute to the knowledge and care provision of vulnerable populations affected by this fungal neglected tropical disease.

## Methods

### Ethics statements

The Institutional Review Board (IRB) of INI-FIOCRUZ approved this study (appreciation number: 53668221.5.0000.5262). A written informed consent form was obtained from the eligible patients who could be reached, while those who were inaccessible were waived, in accordance with the approval granted by the IRB.

### Study design

This is an analytical, observational cohort study, conducted from 1980 to 2020, at the Evandro Chagas National Institute of Infectious Diseases (INI-FIOCRUZ), a reference center for PCM in the endemic area of Rio de Janeiro, Brazil.

### Patients

We performed an active case search on the institutional databases of INI-FIOCRUZ from 1980 to 2020. The sampling strategy selected patients registered under the identification code of PCM (B41) according to the International Classification of Diseases (ICD) in both “definitive diagnosis” and “diagnostic hypothesis” fields, and who presented any of the following terms in their medical records: pregnancy, or pregnant woman, or childbirth, or puerperium, or puerperal woman, or prenatal, or abortion. The next step was the review of the medical records aiming to fill the inclusion criteria, which consisted in the confirmation of the PCM diagnosis according to the Brazilian Guidelines on PCM [2], along with the occurrence of pregnancy shortly before (up to a month) or at admission, during the PCM treatment, or while the post-therapeutic follow-up. In our clinical practice, we use the following strategies to mitigate the risk of patients being lost to follow-up: maintenance of active and regular communication with those who are absent, as well as insurance of regular medication delivery without costs to the patients. After inclusion, we de-identified the medical records to protect patients’ privacy.

### Data analysis

The variables analyzed were (a) socio-demographic and epidemiological data (age, skin color, level of education, marital status, place of residence, professional occupation, and risk activities related to PCM); (b) clinical data (duration of symptoms prior to PCM diagnosis, comorbidities, PCM clinical forms, affected organs, and severity of PCM [2]); (c) information regarding the PCM laboratorial diagnosis (clinical specimens, type and results of the laboratorial methods used, serological titers of specific antibodies against *Paracoccidioides* spp. as detected by double immunodiffusion test–DID [20]); (d) information on the therapeutic management of PCM (prescribed antifungal drugs, dose and treatment duration, adherence, and adverse events); (e) gestational data (timing of the PCM follow-up care when the pregnancy occurred; prenatal information including gestational diseases, intercurrences, vaginal bleeding, co-infections, or a history of abortion; delivery information such as if full-term or premature birth and newborn health parameters such as birth weight and Apgar-5 [21]; and other conditions that could interfere in the gestational health such as smoking, alcohol abuse, illicit drug use and the consumption of other medications during pregnancy); (f) prognostic data (occurrence of complications related to both PCM and pregnancy, as well as maternal-fetal and PCM outcomes). Preterm was defined as babies born alive before 37 weeks of pregnancy are completed [22] and low birth weight as less than 2,500 grams (5.5 pounds) [23].

The information regarding pregnancy, childbirth, and newborn health not available in the medical records of INI-FIOCRUZ were obtained from the public official databases of the Sistema de Informação sobre Nascidos Vivos (SINASC) (Brazilian Ministry of Health’s Information System on Live Births) [24]. We also tried to contact the included patients to reduce the missing data.

### Statistical analysis

The variables obtained from the review of medical records were exported and analyzed using Excel spreadsheets. Frequencies, proportions, and summary measures were calculated.

## Results

**Fig 1:**
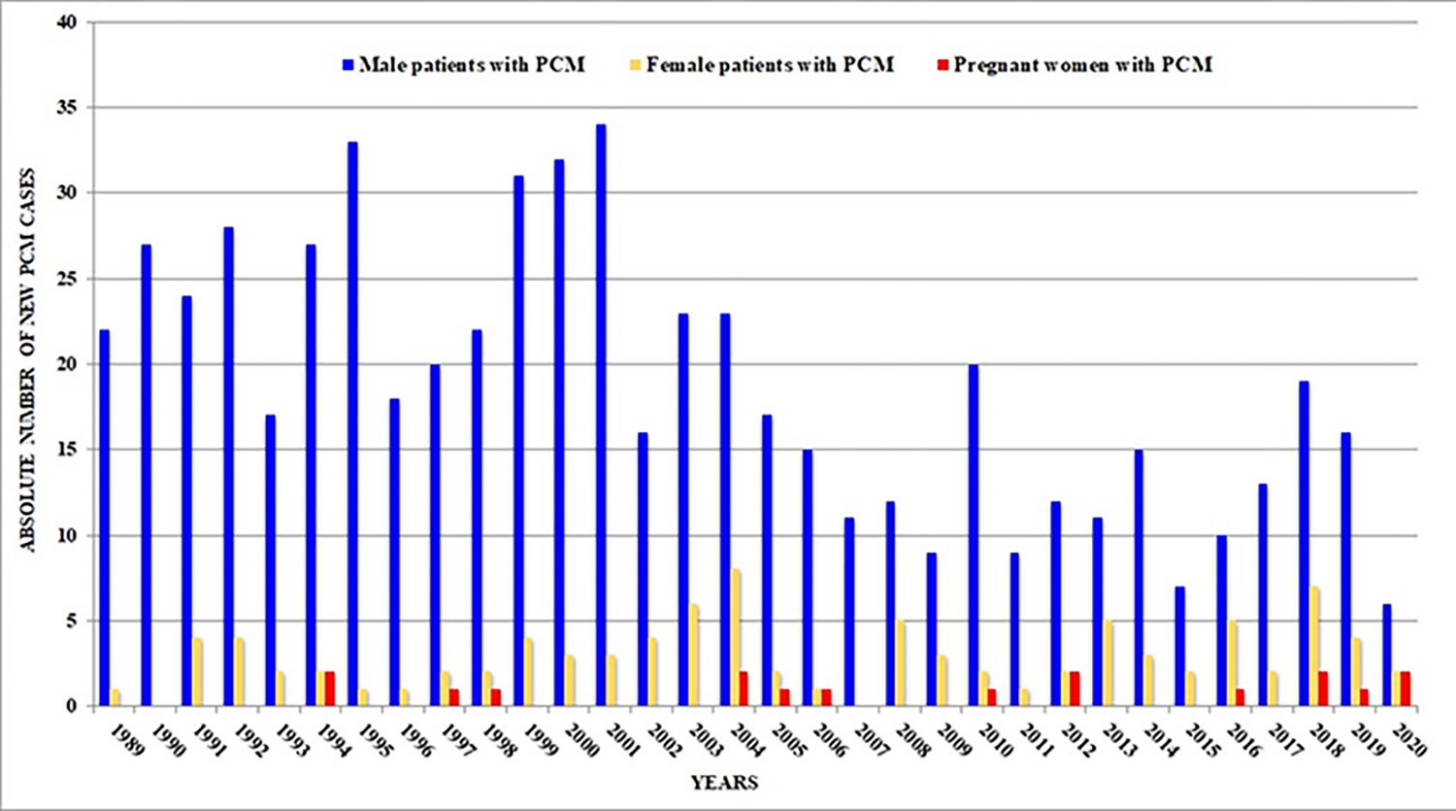
Annual occurrence of pregnancies in patients diagnosed with paracoccidioidomycosis (PCM) at INI-FIOCRUZ from 1989 to 2020. The data sources used for this analysis were obtained from the INI-FIOCRUZ Electronic Patient Record System.

The search conducted in our databases revealed 18 pregnant patients with PCM who received assistance from 1980 to 2020. Fig 1 shows the distribution of these cases alongside the overall number PCM-diagnosed patients followed at INI-FIOCRUZ between 1989 and 2020, which is the timeframe for which we have obtained this information. Therefore, the first pregnant patient of our series, detected in 1986, is not represented in the figure. Among the cases included in this study, 6 (33.3%) were detected between 2016 and 2018.

Among the 18 patients from our study, 16 (88.9%) had one single pregnancy and 2 (11.1%) had two, totalizing 20 pregnancies. Regarding the single pregnancies, three (16.7%) patients were already pregnant either shortly before (up to a month) or at the moment of admission, when PCM was diagnosed. Ten (55.6%) got pregnant during the course of PCM treatment and three (16.7%) during the post-therapeutic follow-up for monitoring the cure of PCM. With regards to the patients who had two pregnancies, one was pregnant shortly before admission and got pregnant again in the post-therapeutic follow-up, while another patient got pregnant twice during the treatment period.

### Socio-demographic and epidemiological aspects

We identified that 10 (55.6%) patients came from urban areas characterized by poor socioeconomic conditions in the *Baixada Fluminense*. This region comprises 13 municipalities and is located in the inner part of the Rio de Janeiro metropolitan region. The median age was 26 years, ranging from 16 to 38 years. The majority of patients was single (n = 12, 66.7%), black (n = 10, 55.6%), and had low levels of education (n = 10, 55.6%). There were no reported occupational activities associated with a risk of PCM infection and the available professional data mostly revealed involvement in commercial activities.

### Clinical features

Regarding the PCM clinical manifestations, the acute juvenile type was the most commonly observed, accounting for 14 (77.8%) cases, while the chronic form occurred in four (22.2%) cases. The median duration from the onset of clinical symptoms to the diagnostic confirmation of PCM was four months (range: 1–24 months).

**Fig 2:**
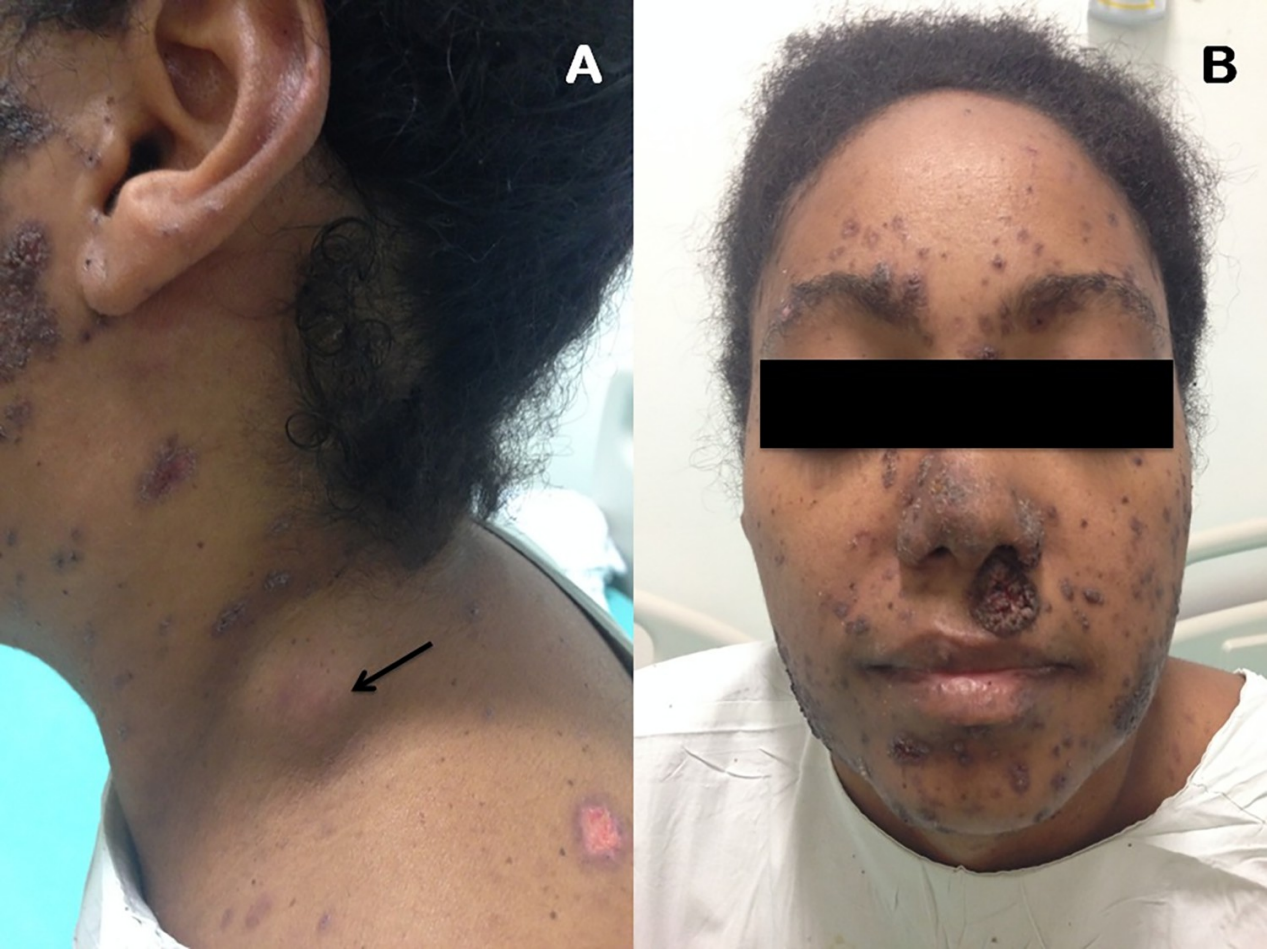
Lymph node enlargement and cutaneous lesions resulting from hematogenous dissemination of *Paracoccidioides* sp. in a patient from this study. A 30-year-old patient, presenting (A) Cervical lymph node enlargement (black arrow) and (B) Papules and ulcerative lesions covered with crusts on the face.

Among the affected organs, the lymph nodes were the most commonly involved, affecting 15 (83.3%) patients, followed by the skin, which was affected in eight (44.4%) cases (Fig 2). Other organs involved were the lungs (n = 6, 33.3%), spleen (n = 3, 16.7%), liver (n = 2, 11.1%), adrenals (n = 2, 11.1%), large intestine (n = 1, 5.6%), and the central nervous system (CNS) (n = 1, 5.6%).

Regarding the severity of PCM cases, there was an overall predominance of moderate (n = 9, 50%) and severe (n = 8, 44.4%) forms, while the single patient with mild disease presented the chronic form. Among the 14 patients with acute PCM, moderate and severe forms were equally distributed, with seven cases, each. Among the four patients with chronic PCM, mild and severe presentations accounted for one case each, while the moderate occurred in two patients. Only one patient from this study had comorbidities. She was a 38-year-old patient with a history of smoking, cardiovascular disease, chronic obstructive pulmonary disease (COPD), and developed a moderate chronic PCM.

### Laboratorial diagnosis of PCM

The PCM diagnosis predominantly relied on clinical specimens obtained from lymph nodes (n = 11, 61.1%), followed by skin or mucosal lesions (n = 7, 38.9%), and sputum samples (n = 2, 11.1%). The main methods performed were direct examination (n = 17, 94.4%) and culture (n = 16, 88.9%) with positive results in 94.1% and 75%, respectively. Histological examination was performed in 11 (61.1%) cases and yielded a positivity rate of 72.7%. Immunological tests (DID) were conducted in all cases and most patients (n = 13, 72.2%) had detectable levels of anti-*Paracoccidioides* antibodies before initiating antifungal treatment, with titers ranging from 1:8 to 1:2,048.

### Therapeutic management and prognosis of PCM

The median duration of antifungal treatment was 17.5 months (range: 6–39 months). The therapeutic regimen usually involved multiple drugs (n = 12, 66.7%), administered in combination or alternately, usually initiating with amphotericin B (AMB) induction therapy (n = 9, 50%). AMB was used in 10 (55.6%) cases. Itraconazole (ITZ) and sulfamethoxazole-trimethoprim (SMZ-TMP) were the main oral drugs prescribed (n = 11, 61.1% and n = 10, 55.6%, respectively) either alone or in combination, to achieve better disease control. Sulfadiazine (SDZ) was prescribed in three (16.7%) cases and fluconazole (FCZ) was associated with SMZ-TMP for a patient with PCM neurological involvement. The first patient identified in the current study was treated with ketoconazole (KTZ) in 1986.

The therapeutic regimens were usually well tolerated, with few cases experiencing important adverse events. Hypersensitivity and kidney dysfunction were observed in one case each, both due to SMZ-TMP and resolved upon drug discontinuation.

Sixteen (88.9%) patients evolved to cure after a median follow-up period of 71 months (range: 12–216 months) of post-therapeutic follow-up. Two (11.1%) patients met the cure criteria, but were lost to follow-up after discontinuing treatment due to the pregnancy. Any patient among those who had their PCM treatment discontinued due to pregnancy did not experience clinical worsening after the drug interruption. All patients presented negative DID results at the time of pregnancy detection, except for patients who were pregnant shortly before admission. There were no relapses during the post-therapeutic follow-up and all patients evolved to serological cure. Two (11.1%) patients had complications related to PCM, both presenting adrenal insufficiency.

### Gestational profile and outcomes

Three (16.7%) patients had complications during pregnancy, including urinary infection, vaginal bleeding, and preeclampsia with HELLP syndrome (hemolysis [H], high levels of enzymes liver [EL] and low platelets [LP]), one case each. In all three cases, the patients were undergoing PCM treatment, either in part (the first case), or throughout the pregnancy, in the other two. The first patient was taking SMZ-TMP and FCZ, which were discontinued upon pregnancy detection. The second patient was initially on ITZ, which was switched to SDZ after pregnancy detection. The third patient was initially on SMZ-TMP, which was discontinued upon pregnancy detection, and after two months required SDZ and subsequently AMB, for a better control of PCM during pregnancy.

Regarding the newborns, we observed complications in five pregnancies: two preterm births, four cases of low birth weight (including a twin pregnancy), and two fetal deaths. Mothers were undergoing PCM treatment at the time of pregnancy detection in all these cases and received drugs such as ITZ and SMZ-TMP during the first and part of second trimesters.

**Fig 3:**
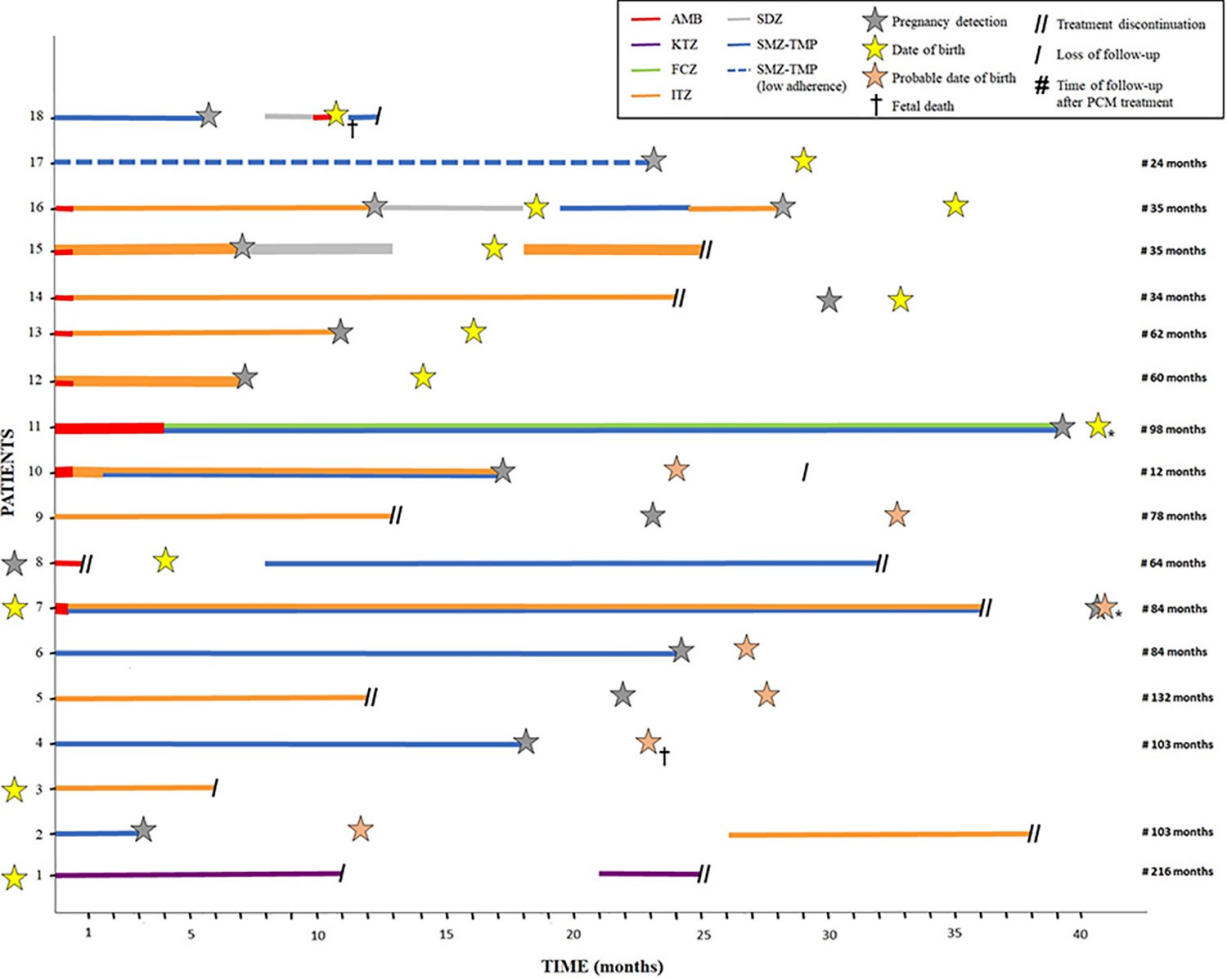
Progression of each case included in the study. The timelines depict the point at which pregnancy was detected during the PCM follow-up care, the timing of delivery (including any fatal outcomes), the prescribed therapeutic regimen with the chronology of the drugs, the duration of treatment, and the length of post-therapeutic follow-up. Asterisks denote two pregnancies that occurred within the expected duration, but could not be represented precisely due to space limitations. Treatment discontinuation, represented by the two bars, was made by the assistant physicians. AMB: amphotericin B; KTZ: ketoconazole; FCZ: fluconazole; ITZ: itraconazole; SDZ: sulfadiazine; SMZ-TMP: sulfamethoxazole-trimethoprim.

Table 1 presents a compilation of the main features observed in the patients with PCM included in this study. Table 2 summarizes the data obtained from SINASC related to the pregnancy, delivery, and newborn health. Furthermore, Fig 3 depicts a timeline for each case herein studied, illustrating the point at which pregnancy was detected during PCM follow-up care, along with the therapeutic regimen for PCM, the chronology of drugs, the treatment duration, and the post-therapeutic follow-up period.

**Table 1:** Main clinical, laboratory and therapeutic data regarding PCM observed in the cases analyzed in this study.

PCM case	Age	Year of PCM diagnosis	Year of PREG	PCM status on PREG #	Time of symptoms (months)	Method of PCM diagnosis (clinical specimen)	DID result (titers) at admission	PCM clinical form	PCM grade of severity✝	Time of treatment (months)	PCM outcome
**1**	21	1986	1986	AA	6	DE / Cult / HP (lymph node biopsy)	Positive (1:1,024)	Acute	Moderate	15	Cure
**2**	22	1992	1994	DT	24	DE / Cult (sputum and skin biopsy)	Negative	Acute	Moderate	15	Cure
**3**	37	1994	1994	AA	15	DE / Cult / HP (oral mucosa scraping, skin and lymph node biopsies)	Positive (1:128)	Chronic	Moderate	6	LFU
**4**	33	1997	1997	DT	18	DE / Cult (sputum)	Positive (1:16)	Chronic	Severe	18	Cure
**5**	30	1998	1998	FU	24	DE (skin biopsy)	Negative	Chronic	Mild	12	Cure
**6**	20	2004	2006	DT	3	DE / Cult (lymph node aspirate)	Positive (1:16)	Acute	Moderate	24	Cure
**7.1**	18	2004	2004	AA	4	DE / Cult (lymph node aspirate)	Positive (1:8)	Acute	Moderate	36	Cure
**7.2**	//	//	2007*	FU	//	//	Negative	//	//	//	//
**8**	38	2004	2004	AA	5	DE / Cult (oral mucosa scraping)	Positive (1:32)	Chronic	Moderate	25	Cure
**9**	25	2004	2005	FU	3	DE / Cult (skin exudate)	Negative	Acute	Severe	13	Cure (S)
**10**	17	2008	2010	DT	3	DE / Cult (lymph node aspirate)	Positive (1:16)	Acute	Moderate	17	Cure
**11**	27	2009	2012	DT	2	DE / Cult (lymph node aspirate)	Positive (1:16)	Acute	Severe	39	Cure
**12**	24	2012	2012	DT	3	HP (lymph node biopsy)	Negative	Acute	Moderate	7	Cure
**13**	28	2016	2016	DT	NA	HP (lymph node biopsy)	Negative	Acute	Severe	11	Cure (S)
**14**	30	2017	2020	FU	2	DE / HP (lymph node biopsy)	Positive (1:128)	Acute	Severe	24	Cure
**15**	25	2018	2018	DT	13	DE (lymph node aspirate) / HP (colon biopsy)	Positive (1:2,048)	Acute	Severe	20	Cure
**16.1**	16	2018	2019	DT	12	DE / HP (lymph node biopsy)	Positive (1:2,048)	Acute	Severe	27	Cure
**16.2**	//	//	2021*	DT	//	//	Negative	//	//	//	//
**17**	27	2018	2020	DT	3	DE / Cult (lymph node aspirate)	Positive (1:256)	Acute	Moderate	23	Cure
**18**	28	2018	2018	DT	1	DE / Cult/ HP (skin biopsy)	Positive (1:16)	Acute	Severe	9	LFU

PCM: paracoccidioidomycosis; PREG: pregnancy; //: same as above

*: second pregnancy

#: moment of the PCM follow-up care when the pregnancy was detected; AA: at admission; DT: during treatment; FU: during the post-therapeutic follow-up; NA: not available; DE: direct examination; Cult: culture; HP: histopathology; DID: double immunodiffusion

✝: Based on the Brazilian guidelines for the clinical management of PCM [2]; LFU: lost of follow-up; (S): sequelae (adrenal impairment). Case 14 is represented in Fig 2.

**Table 2:** Main data of the pregnancy, delivery, and newborns birth parameters, from patients with paracoccidioidomycosis included in this study.

Newborns	Way of childbirth	GA (weeks)	NB sex	Birth weight (grams)	Apgar-5	Complications of pregnancy / delivery	ICU need
**NB 1**	NA	NA	NA	NA	NA	NA	NA
**NB 2**	NA	NA	NA	NA	NA	NA	NA
**NB 3**	Vaginal	38	M	3,450	8	No	No
**NB 4**	NA	NA	NA	NA	NA	Fetal death	NA
**NB 5**	NA	NA	NA	NA	NA	NA	NA
**NB 6.1** *	Cesarean	38	F	2,185	9	Low birth weight	No
**NB 6.2** *	Cesarean	38	F	1,655	9	Low birth weight	No
**NB 7.1**	NA	NA	NA	NA	NA	NA	NA
**NB 7.2**	NA	NA	NA	NA	NA	NA	NA
**NB 8**	Vaginal	NA	F	2,530	9	No	No
**NB 9**	NA	NA	NA	NA	NA	NA	NA
**NB 10**	NA	NA	NA	NA	NA	No	No
**NB 11**	Vaginal	NA	F	NA	NA	Urinary tract infection	No
**NB 12**	Cesarean	39	M	3,060	9	No	No
**NB 13**	Cesarean	40	M	3,074	9	No	No
**NB 14**	Vaginal	38	M	2,936	10	No	No
**NB 15**	Cesarean	39	F	3,240	10	Vaginal bleeding	No
**NB 16.1**	Vaginal	37	M	2,160	9	Preterm birth / Low birth weight	No
**NB 16.2**	Vaginal	32	M	1,584	9	Preterm birth / Low birth weight	Yes
**NB 17**	Vaginal	40	M	3,615	9	No	No
**NB 18**	Cesarean	23	M	630	8	Preeclampsia with HELLP / Fetal death	Yes

NB: Newborn (numbered in correspondence to their mothers, the patients listed in Table 1)

*: twin pregnancy; GA: Gestational Age; Apgar-5: score used to assess the clinical status and the vital parameters of the newborn after 5 minutes of birth (scale 0–10). ICU: Intensive Care Unit; NA: not available; F: Female; M: Male; Low birth weight: < 2,500 grams; Preterm birth: born alive before 37 weeks of pregnancy are completed; HELLP: syndrome associated with severe preeclampsia composed by hemolysis, elevated liver enzymes, and low platelets.

Source: SINASC (Brazilian Ministry of Health’s Information System on Live Births).

## Discussion

The occurrence of PCM during pregnancy is rare, but there has been a recent upward trend in the state of Rio de Janeiro. This increase can be a consequence of anthropogenic epidemiological changes leading to the emergence of acute juvenile PCM in this region [6]. Indeed, as susceptible populations (especially young individuals) are more exposed to this fungal infection, pregnant women are at a higher risk of developing disease presumably due to a reduced immune response during gestation [2]. The socio-demographic and epidemiological profiles of the cases herein reported highlight the vulnerability of this population. The majority of cases involve young black women with low educational attainment, residing in economically disadvantaged peripheral urban areas of the *Baixada Fluminense*, which was not traditionally recognized as an endemic area for PCM in the state of Rio de Janeiro until the last few years. In this region, an outbreak of acute PCM has been recently reported [6], which coincides with the observed predominance of PCM cases during pregnancy detected in the last five years of the study period.

As expected, the acute form of PCM was the predominant presentation in this study, aligning with previous literature reports and considering the young age of the patients [9,10,13,14]. This clinical presentation is characterized by an invasive pattern, often moderate to severe, involving vital organs and leading to serious sequelae in some cases. Although it has been reported that acute PCM is typically diagnosed within a few weeks of symptoms onset [2], we observed a considerable delay between the initial clinical symptoms and the confirmation of PCM diagnosis, resulting in postponed treatment that may have contributed to increased severity. This delay is probably due to gaps in providers’ knowledge regarding acute PCM, which is traditionally rare [2,25]. Notably, the high incidence of persistent lymph node involvement among the patients in our study highlights a significant clinical finding that should raise awareness among healthcare professionals. It is crucial that they consider PCM as a potential diagnosis when evaluating young patients from urban endemic areas to avoid misdiagnosis.

We noticed that the most used laboratory techniques to diagnose PCM were the conventional ones, which yielded a high rate of positive results, as previously reported [26]. The direct examination of lymph node specimens demonstrated excellent performance, and there was a high reactivity in serology using the simple and minimally invasive DID test [27]. Therefore, diagnostic suspicion should be a priority, before considering more advanced laboratorial diagnostic methods.

Treating PCM during pregnancy poses significant challenges. The need for therapeutic induction with AMB, as well as drugs suspension, replacement or combination highlights the complexities involved. While there are limited safe pharmacological options to treat PCM in pregnant women, such as AMB and SDZ [2,17], the most common issue observed in this study was the occurrence of pregnancy during PCM treatment. Over half of the pregnancies occurred at this stage, potentially exposing the fetus to teratogenic drugs, particularly ITZ and SMZ-TMP, the two main oral drugs used to treat PCM [2]. All complications observed in the newborns of our study were related to pregnant women receiving drugs such as ITZ and SMZ-TMP during the first and second trimesters. These drugs present theoretical concerns about possible teratogenicity associated with first-trimester exposure, including congenital malformations and abortion [28–30]. This is an important concern, particularly in the context of the emergence of acute PCM, as young women are in their reproductive age and ITZ may reduce the effectiveness of contraceptives, thereby increasing the risk of pregnancy during the prolonged treatment period of this systemic mycosis. Therefore, in the context of the emergence of acute juvenile PCM in this population, we emphasize the relevance of effective communication between healthcare professionals and these young patients, to reinforce the need to use alternative contraceptive methods, especially barrier methods, to mitigate the risk of pregnancy during PCM treatment. In routine consultations with young women undergoing long-term antifungal treatment, it is essential to inquire about the timing of their last menstrual period as part of their medical history. Although not yet cured, the patients who had their treatment interrupted due to pregnancy did not experienced clinical worsening of PCM, even those who still were at the beginning of treatment. It is noteworthy that these latter had at least three months of treatment or had received treatment induction with AMB, supporting the well-known effectiveness of the available drugs against *Paracoccidioides* spp. and to control clinical disease [17].

Gestational complications have been reported in patients with PCM, including placental involvement, prematurity, and maternal death [9,12]. Experimental murine models have also shown associations between PCM and adverse outcomes, such as abortions and low birth weight [18,19]. While these studies using experimental models infected with *Paracoccidioides* spp. suggest a direct role of the fungal infection in gestational outcomes, previous complications reported in pregnant women with PCM may have been influenced by other factors, such as the use of teratogenic drugs and other infections with congenital repercussions, like syphilis and toxoplasmosis. In the present study, all gestational complications, both maternal and fetal, occurred in patients with PCM who did not presented comorbidities and were undergoing antifungal treatment, suggesting the possible role of the antifungal drugs leading to these outcomes.

Among the comorbidities that pose a higher risk of complications in PCM, infection with the human immunodeficiency virus (HIV) is particularly noteworthy [31]. Finamor and collaborators reported a case of severe acute PCM with ocular and neurological involvement in a pregnant woman with AIDS, which resulted in maternal death [10]. In our study, we did not identify any cases of co-infection between *Paracoccidioides* spp. and HIV, but we identified a case of severe acute PCM with neurological involvement. In this particular case, the pregnancy occurred while the patient was undergoing PCM treatment, which was subsequently discontinued upon pregnancy detection. Although the single complication during this pregnancy was a urinary infection, the baby died at the age of 10 months due to pneumonia of unknown etiology.

Some limitations of this study are worthy to mention such as missing data, what is expected for retrospective studies, and the small numbers of patients evaluated, as PCM in pregnant women is rare. The authors performed a descriptive study aiming to report the first case series of a rare condition published so far. In this context, analytical evaluation of data was not possible, thus it was difficult to establish whether the pregnancy complications were certainly caused by the specific drug used to treat PCM, if they were a direct effect of the fungal infection or other possible undiagnosed conditions. Further research comparing PCM clinical data between pregnant and non-pregnant women is needed to better understand underlying mechanisms and potential contributing factors to the gestational complications in pregnant women with PCM.

## Conclusions

Our findings show the increasing number of cases of PCM during pregnancy in the endemic area of Rio de Janeiro and emphasize the need for public health measures to raise awareness of this neglected disease among healthcare professionals. It is crucial to promote early diagnosis, recommend effective forms of birth control, including both pills and barrier methods, and inquire about the menstrual periods for women of childbearing potential under PCM treatment. By prioritizing the measures above mentioned, we could mitigate the occurrence of pregnancy during PCM treatment, improve management and outcomes for pregnant women with PCM, and protect the health and well being of both mother and children.

## Supporting information

S1 STROBE Checklist STROBE checklist. (DOCX)
